# Mechanisms of Toxicity of Industrially Relevant Silicomanganese Dust on Human 1321N1 Astrocytoma Cells: An In Vitro Study

**DOI:** 10.3390/ijms20030740

**Published:** 2019-02-10

**Authors:** Yke Jildouw Arnoldussen, Torunn Kringlen Ervik, Johanna Samulin Erdem, Ida Kero, Mina Baarnes Eriksen, Vidar Skaug, Shanbeh Zienolddiny

**Affiliations:** 1 Department of Biological and Chemical Work Environment, National Institute of Occupational Health, Pb 8149 Dep., N-0033 Oslo, Norway; yke.j.arnoldussen@stami.no (Y.J.A.); torunn.ervik@stami.no (T.K.E.); johanna.samulin-erdem@stami.no (J.S.E.); mina.eriksen@stami.no (M.B.E.); vidar.skaug@stami.no (V.S.); 2 Department of Industrial Process, Technology SINTEF Materials and Chemistry, PB 4760, N-7465 Trondheim, Norway; ida.kero@sintef.no

**Keywords:** neurotoxicity, silicomanganese, occupational, nanotechnology

## Abstract

Tremendous efforts are applied in the ferroalloy industry to control and reduce exposure to dust generated during the production process, as inhalable Mn-containing particulate matter has been linked to neurodegenerative diseases. This study aimed to investigate the toxicity and biological effects of dust particles from laboratory-scale processes where molten silicomanganese (SiMn) was exposed to air, using a human astrocytoma cell line, 1321N1, as model system. Characterization of the dust indicated presence of both nano-sized and larger particles averaging between 100 and 300 nm. The dust consisted mainly of Si, Mn and O. Investigation of cellular mechanisms showed a dose- and time-dependent effect on cell viability, with only minor changes in the expression of proteins involved in apoptosis. Moreover, gene expression of the neurotoxic biomarker *amyloid precursor protein* (*APP*) increased, whereas APP protein expression decreased. Finally, induction of gap junctional intercellular communication (GJIC) increased with higher doses and correlated with the other endpoints. Thus, the effects of SiMn dust on 1321N1 cells are highly dependent on the dose of exposure and involves changes in APP, apoptosis-related proteins and intercellular communication.

## 1. Introduction

Silicomanganese (SiMn) is important for the production of steel and is used as an alloying element in almost all types of steel. Characterization of the fumes collected in a SiMn plant indicated that the aerodynamic diameter of the particles ranged from 7 nm to 10 µm [1]. Among these, 78% of the particles were classified as ultrafine (aerodynamic diameter < 100 nm), whereof 40% had an aerodynamic diameter below 30 nm. Moreover, the smallest fractions of the fume had a larger dominance of Si than the larger fractions and the ultrafine sized particles contained a significantly smaller amount of Mn than the larger particles [1]. Similar dust was generated in a more controlled laboratory setting by Kero et al. and Ma et al. [2,3]. Characterization of such laboratory dust showed that the particles are oxides and very often amorphous. Typically, the primary units are spheres that may agglomerate and aggregate. The dust generated from SiMn production has been shown to be quite different from other ferroalloy processes, such as ferrosilicon [1,4,5].

It is known that a fraction of the particles that deposit in the respiratory tract after inhalation can translocate across the epithelial linings and enter the circulation [6]. Moreover, ultrafine inorganic particles may translocate to the brain by passage through the olfactory epithelium and olfactory nerve [7,8]. Uptake of particles and their ability to cross the blood-brain barrier (BBB) depends highly on their size, including the amount of aggregation, and other factors such as charge and coating from contact with biological fluids. Accumulation of Mn in the brain and damage to the nervous system of workers as a result of exposure to Mn under occupational settings may appear [9,10,11,12]. Moreover, translocation of Mn from the respiratory system to different areas of the brain upon inhalation was shown in a study using rodents that inhaled manganese oxide particles [13]. Manganese dioxide nanoparticles were shown to induce toxicity and apoptosis in human neuronal cells [14]. Although distribution of Mn particles was observed in neurons, Mn concentrations are thought to be higher in astrocytes than neurons after exposure [15]. Currently, the mechanisms underlying astroglial dysfunction as a result of Mn exposure are not completely understood. Mn exposure of primary mouse astrocytes and a human astrocytic cell line showed that Mn exposure induced the release of proinflammatory cytokines and exacerbated the inflammatory response [16]. There is also concern on the potential of silica particles to induce inflammatory responses in the brain. A study using amorphous SiO_2_ nanoparticles showed that exposure of neuronal cells decreased cell viability, induced apoptosis by activating the p53-mediated signaling pathway and disturbed the cell cycle [17]. It was also shown that the amorphous SiO_2_ nanoparticles induced similar responses in glioblastoma cells [18].

Astrocytes are the most abundant glial cells of the central nervous system and have been shown to be important in the regulation of some brain immunological responses [19]. They regulate the functions of neurons and are involved in the neuroinflammatory response, including neurodegenerative diseases. Their activation occurs in most brain pathologies and is associated with altered gene expression, which may exert inhibitory effects on the central nervous system [20]. By using an in vitro model for the BBB, SiO_2_ nanoparticles induced loss of tight junctions and cytoskeleton arrangement, and increased the inflammatory response [21]. Here, we investigated toxicological effects of molten, air-exposed SiMn dust from a laboratory scale process, using an astrocytoma-derived cell line as model. Astrocytes are important for communication between cells in the brain and deletion of astroglial connexins weakens the BBB [22]. To analyze the effects of SiMn dust on the BBB, gap junctional intercellular communication (GJIC) was investigated. Moreover, changes in the expression of the neurotoxicity marker, amyloid precursor protein (APP), and regulation of proteins involved in apoptosis were studied.

## 2. Results

### 2.1. Characteristics of the Dust

**Figure 1:**
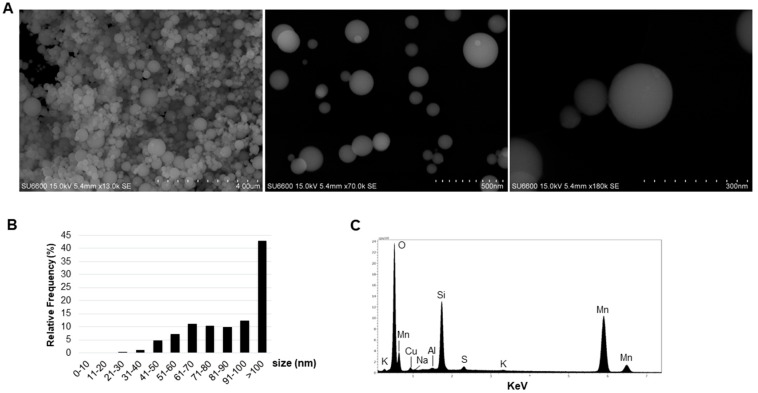
Characterization of dry SiMn dust by SEM. (**A**) Representative SEM images. (**B**) The diameter (nm) of the dust particles was measured and the relative frequency in percentage is shown for the different size groups with specific focus on particles ≤100 nm (*n* = 252). (**C**) Energy-dispersive X-ray spectrum showing the elemental content of the dust particles.

Detailed characteristics of the SiMn dust produced in the laboratory have been published previously [2]. Briefly, the dust was produced at 1500 °C and collected on a filter. The dust was stored at ambient atmosphere. Investigation of the dust by scanning electron microscopy (SEM) showed that it consisted of primary spherical particles of various sizes (Figure 1A). Measurement of the diameter of the particulate matter indicated that 58% of the investigated primary particles were between 20 and 100 nm in size (Figure 1B). The relative frequency for the size groups 31–40, 41–50 and 51–60 nm was 1.2%, 4.8% and 7.1%, respectively. For each of the other size groups, including 61–70, 71–80, 81–90 and 91–100 nm, the relative frequency was approximately 10% (Figure 1B). Analysis of the elemental contents of the dust collected on the filter indicated a main content of Si, Mn and O, in addition to trace elements of K, S, Al, Na and Cu (Figure 1C). The Cu intensity found in the spectrum arises from the TEM grid. The spectrum was obtained by measuring over almost the entire area seen in Figure 1A.

**Figure 2:**
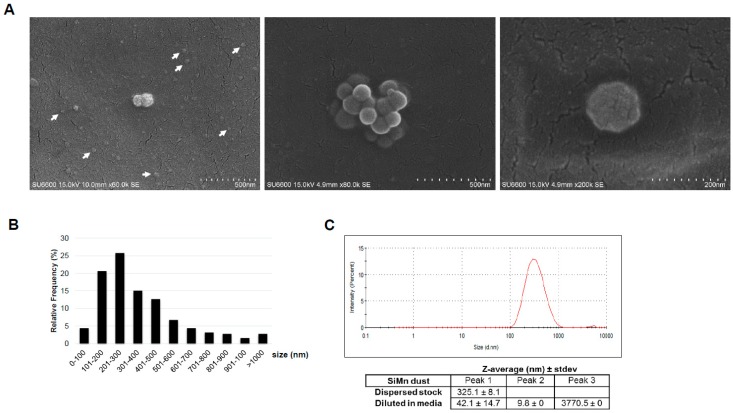
Characterization of dispersed SiMn dust. (**A**) A volume corresponding to 100 µg dust was taken from a 1 mg/mL stock dispersed in 0.05% BSA and filtered through a 47 mm Whatman Nuclepore polycarbonate filter with 15 nm pore size. The dust was investigated by SEM and representative images are shown. Arrows point to nano-sized particles that were difficult to visualize due to the platinum coating. (**B**) The diameter (nm) of the dust particles was measured and the relative frequency in percentage is shown for the different size groups (*n* = 252). (**C**) Size distribution and average hydrodynamic diameter of the dispersed SiMn dust. One mL of the dispersed SiMn stock solution was used for DLS measurements to obtain the size distribution and average hydrodynamic diameter of the dust. 10 cycles were run. The graph showing the size distribution is representative of one measurement over 10 cycles. The Z-average from three independent dispersed batches is shown ± standard deviation (SD).

To expose the cultured cells, the dust was dispersed in solution containing bovine serum albumin (BSA) and subsequently, characterization of the dispersed dust was performed by SEM, in addition to dynamic light scattering (DLS). Representative SEM images of some of the particle structures of dispersed dust in solution are shown in Figure 2A. Due to the platinum coating of the dispersed specimens for SEM, the smallest particles were difficult to visualize; however, they were certainly present and support the obtained results from analysis of the dry dust, as shown in Figure 1. Size measurements showed that most of the particles >100 nm were in the size ranges of 101–200 nm (20.6%), 201–300 nm (25.8%), 301–400 nm (15.1%) and 401–500 nm (12.7%) (Figure 2B).

Measurements of the hydrodynamic size by DLS indicated that the majority of the particles in the dissolved dust had an intensity weighted mean hydrodynamic size (Z-average) of 325.1 ± 8.1 nm with a stable size distribution (Figure 2C). For investigation of the dusts’ behavior in cell culture media, the size distribution and size stability toward agglomeration of SiMn was determined (Figure 2C). To this end, three peaks were detected consisting of particles with the following sizes: 42.0 ± 14.7 nm, 9.8 ± 0.0 nm and 3770.5 ± 0.0 nm.

### 2.2. Cellular Responses of Exposure to SiMn Dust

**Figure 3:**
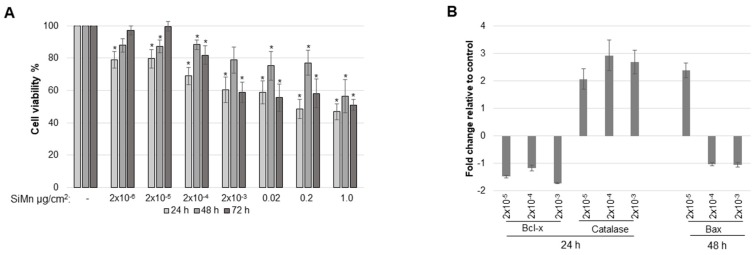
SiMn-induced cytotoxicity is dose and time dependent and affects apoptosis-related proteins. (**A**) Astrocytoma cells were grown and exposed to sham or to SiMn dust at the indicated concentrations for 24, 48 and 72 h before measurement of cellular cytotoxicity. Cell viability of sham-treated cells was set to 100%. An average of three independent experiments in triplicate is shown. (**B**) The expression levels of 35 proteins related to or involved in apoptosis were analyzed using the Proteome Profiler™ Human Apoptosis Array Kit. The results from three independent experiments were quantified and significant changes (*p* ≤ 0.05) in fold change related to control-exposed cells are shown in the bar graphs. A fold increase or decrease of 1.5 was set as cut-off and only the results for those proteins are shown. Error bars: standard error (SE).

Toxicity assays with SiMn dust were performed. Exposure of the cells to increasing concentrations of dust indicated a dose- and time-dependent effect on cell viability (Figure 3A). The lowest doses (2 × 10^−6^ and 2 × 10^−5^ µg/cm^2^) induced a reduction of ~20% in cell viability after 24 h. However, after 48 and 72 h this effect was reversed. At higher doses (2 × 10^−4^–1 µg/cm^2^), a reduction in cell viability was present at all analyzed timepoints (Figure 3A).

To investigate if proteins involved in apoptosis were affected by SiMn exposure, a multiple protein array containing proteins involved in the intrinsic and extrinsic apoptotic pathways was used. The intensities of the protein spots on the arrays were quantified and fold changes for each protein compared to control exposed cells are presented as a heatmap (Table S1) with changes of more than 1.5-fold presented graphically in Figure 3B. B-cell lymphoma extra-large (Bcl-xl), an anti-apoptotic protein, is significantly downregulated after 24 h (Figure 3B). In addition, catalase, an enzyme important for protecting cells from oxidative damage by reactive oxygen species (ROS), is significantly upregulated at the same time. After 48 h pro-apoptotic Bax increased more than 1.5-fold, but only with 2 × 10^−5^ µg/cm^2^ (Figure 3B). Of interest is also cleaved caspase-3 that is increased ~1.3-fold after 48 h with 2 × 10^−5^ and 2 × 10^−4^ µg/cm^2^ (Table S1). Moreover, the cell cycle regulator phospho-Rad17 is significantly decreased by 1.45-fold with 2 × 10^−5^ µg/cm^2^ after 48 h (Table S1).

**Figure 4:**
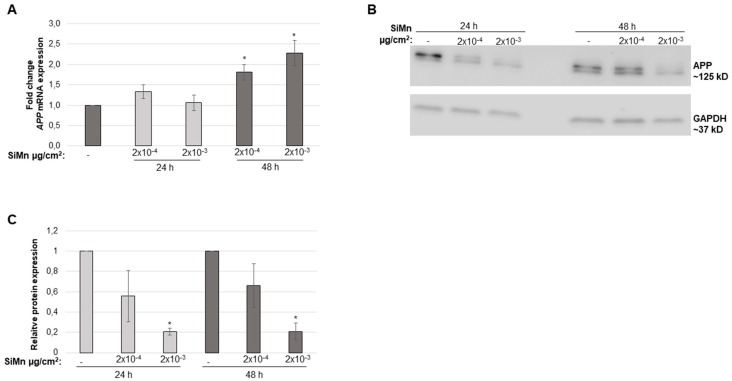
Expression levels of APP after exposure to SiMn. Astrocytoma cells were exposed to SiMn for 24 and 48 h with the indicated doses. mRNA expression was investigated by qPCR. (**A**) *APP* mRNA expression levels. An average from three independent experiments in triplicate is shown. (**B**) APP protein expression levels. Representative western blot images are shown. (**C**) Quantification of APP protein expression. An average from three independent experiments is shown. Values represent the mean ± standard error (SE). * *p* ≤ 0.05 between exposed cells and the sham-treated control. Error bars: SE.

**Figure 5:**
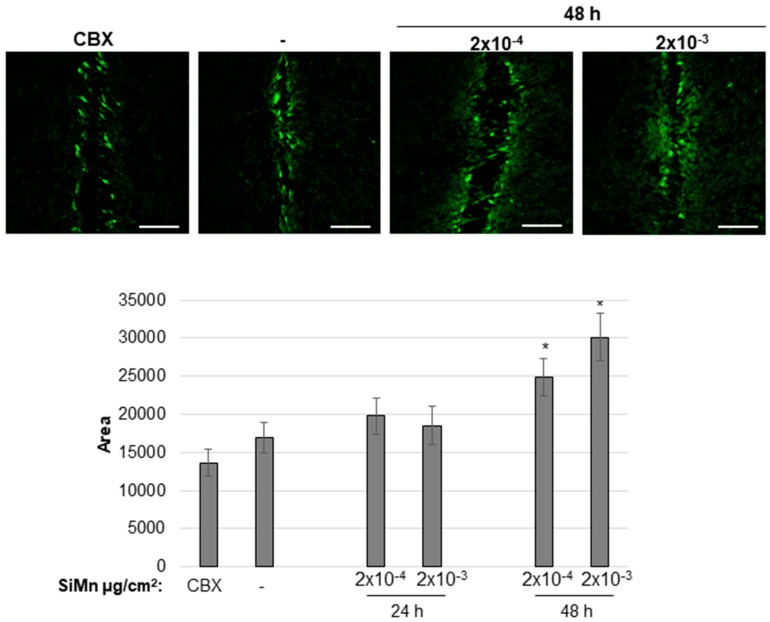
Gap junctional intercellular communication increases by SiMn dust exposure. Astrocytoma cells were grown on coverslips and exposed to dispersion media alone as a control or to the indicated doses of dispersed dust for 24 and 48 h. Carbenoxolone (CBX), an inhibitor of GJIC, was included in one well for each experiment as a control for dye uptake solely by the cut in the cell layer. Scrape loading was performed using Lucifer Yellow. Confocal microscopy was used to detect fluorescence and the levels of gap junctional intercellular communication were determined by means of the area of dye-coupled cells. Representative images are shown. Scale bar: 200 µm. Quantification of three independent experiments is shown. Bars: SE. *: *p* ≤ 0.05 indicates a significant difference between exposed cells and the corresponding sham-treated control.

Based on results from analysis of viability and apoptosis, the two doses (2 × 10^−4^ and 2 × 10^−3^ µg/cm^2^), were chosen for further work. Expression analysis of *APP* indicated no significant differences after 24 h exposure with the different SiMn dust concentrations (Figure 4A); however, after 48 h a significant increase is observed. Conversely, APP protein levels decreased with increasing dose (Figure 4B,C). Moreover, effects of exposure to SiMn dust on gap junctional intracellular communication (GJIC) were analyzed. GJIC in the 1321N1 cells was significantly increased following 48 h of exposure to SiMn dust; however, no significant changes were detected after 24 h of exposure (Figure 5).

## 3. Discussion

In this study, low doses of SiMn dust were used to investigate toxic effects on 1321N1 cells, and to assess molecular mechanisms of brain inflammation such as cellular communication and expression of APP. The use of astrocyte-derived cells is important and practically feasible in line with not using animal studies and is the first step to evaluate responses of astrocytes to occupational relevant dust particles. The doses used in this study are more relevant as they were considerably lower than those used in previous in vitro studies. In a production facility, dust is generated through different processes. These processes are either thermal or mechanical in nature. There are reasons to believe that the smallest particles present in a plant are generated thermally in situations where liquid metal comes into contact with ambient air. However, even the thermal processes can vary considerably in terms of parameters such as temperature and metal flow turbulence. Such parameters will in turn affect the properties of the dust particles, including the oxidation state. Thomassen et al. have shown that the oxidation state of Mn in Mn-containing oxide dust determines the solubility of the particles [23]. Results from different animal studies indicate that the solubility of inorganic manganese compounds can influence the delivery of manganese to target organs such as the brain. This in turn will then affect the possible induction of neurotoxicity, but the effect of the various oxidation states of the manganese is currently not well understood [24].

The ventilation system in a plant is typically more effective in removing dust from certain operations than others. This includes suction hoods for stationary and continuous operations that effectively remove dust and other airborne pollutants. Fugitive emissions from batch-wise processes, such as casting, where vehicle access is crucial to ensure safe and timely operation are often more difficult to abate. On top of these considerations, industrial exposure of dust is never completely isolated from other airborne pollutants, including gases, vehicle exhaust, etc. Industrial activities and burning of fossil fuels leads to enhancement of manganese concentrations in the environment, including soil and water. However, the present study does not deal with the effect and release of silicomanganese in the environment.

In this study, the dust particles were produced in a manner that is thought to simulate conditions for non-turbulent casting in dry atmosphere. This allowed for less variation in terms of particle properties and ensured that dust was not contaminated by other agents. It is very important, however, to keep in mind that the type of dust used here only represents one of many different dust types that may be encountered in a SiMn smelter. Since the dust has been collected in a filter and stored at ambient conditions for some time, the aggregation of dust particles in this study may be different from the freshly generated particles dispersed in the air near the molten metal sources in a plant. Moreover, the relatively weak bonds of aggregates are most likely broken in the sample preparation step where the particles are dispersed in liquid solution for cell culture experiments. The effects of the liquid solution on surface effects and/or aggregation has to date not been evaluated.

The size and elemental content of the particles in the dust is an important determinant for cellular outcome. Investigation of the dry dust indicated that 58% of the particles were ≤100 nm in diameter, reflecting presence of ultra-fine particles in the SiMn dust. The smallest nano-sized particles may due to their small size and high surface area have easier access to organs and tissues in the body, and have a larger impact on cell viability than larger sized particles [21]. Presence of nano-sized particles in the dust was also shown by DLS on the cell culture media containing particles that cells were exposed to. Studies show that nano-sized particles may disturb cellular functions in a larger degree than particles >100 nm [21]. Elemental determination by EDX of the dust indicated presence of mainly Si, Mn and O. Occupational exposures have shown that Mn-containing fumes can cause neurological and neurobehavioral symptoms [25,26]. In general, there are few in vitro studies using Si- and Mn-containing dusts, and cellular effects of silica on glial cells are limited, as reviewed in [27]. Astrocytes play an important role in maintaining the BBB, in regulating neurons and are involved in neuroinflammatory responses [28,29,30]; therefore, a well-established astrocyte-derived astrocytoma cell line was used in the present study. Moreover, astrocytes are increasingly linked to neurodegenerative diseases [31,32,33]. Several studies have shown that particles may translocate through the BBB [34,35,36,37], emphasizing the importance to gain knowledge of the actual mechanisms that are part of a possible neurotoxic response. Currently, data is limited on the actual amount of particles that may translocate to the brain through the various routes, either involving the pulmonary or olfactory pathways. Oxidative stress and activation of oxidative-stress sensitive kinases have been implicated in Mn-induced adverse effects, and Mn has been shown to enter the mitochondrial matrix [38,39,40]. In regard to silica, a study found that surface-modified mesoporous silica nanoparticles entered the brain crossing the BBB in vivo [41]. Moreover, it was shown that amorphous silica nanoparticles induced apoptosis, oxidative stress and autophagy in glioblastoma cells [18], activated astrocytes and induced loss of tight junctions and cytoskeleton arrangement [21]. Hence, both Si and Mn in the dust used in the present study may be important for the observed effects on the endpoints investigated. This is in line with a recent study of industrial relevant silica dust in 1321N1 cells that showed a dose- and time-dependent effect on cell viability [42]. However, these studies are carried out in astrocytoma cells and it would be of interest to compare the results obtained in astrocytoma cells, to healthy human glial brain cells to shed a light on potential differences between healthy and diseased glial cells as the cells may respond differently to the exposures.

The observed restoration of cell viability with the lowest doses in the present study shows that the effects may be acute and transient. For the higher doses, viability decreased with time. The apoptosis protein array indicated small changes which may not be of biological relevance for cellular outcome for most proteins. The downregulation of the anti-apoptotic protein Bcl-xl, with in parallel an increase in catalase after 24 h, may suggest that the cells try to recover from the acute and transient inhibitory effects of exposure to SiMn as these two proteins are important for protecting cells from oxidative damage and from cell cycle arrest. Although Bax and cleaved caspase-3 are increased with 2 × 10^−5^ µg/cm^2^ after 48 h, and phopho-Rad17, a regulator of the cell cycle, is decreased, this may be in favor of cell death. However, with this dose, cell viability is restored to 100% after 72 h, and thus it is likely that the regulation of proteins at the 48 h time point is transitory. Other studies that use much higher doses have a much more robust effect on proteins involved in apoptosis. This is exemplified by a study in PC-12 neuronal cells that were exposed to amorphous SiO_2_ nanoparticles with doses up to 200 µg/mL. Here, an increase in expression of phospho-p53, p21, Gadd45 and Bax, but not of anti-apoptotic Bcl-2, was observed [17].

Amyloid precursor protein (APP) is expressed by astrocytes, is increased upon cellular stress and gives rise to the neurodegenerative marker Amyloid β. In this study, *APP* expression increased, whereas APP protein decreased. A similar response, an increase in *APP* mRNA and a reduction in APP protein, was observed in neuroblastoma cells that were irradiated with ultraviolet (UV) light [43]. APP cleavage gives rise to Amyloid β which is found in the brain of patients with Amyloid β-related disorders and an increase in Amyloid β is associated with chronic inflammatory responses in the brain [44]. Also, there is increased evidence that astrocytes secrete significant quantities of Amyloid β [45]. A study in neuroblastoma cells showed an increase in Amyloid β expression after exposure to amorphous SiO_2_ nanoparticles which also reduced cell viability and increased apoptosis [46], supporting the results obtained with the 1321N1 cells. In addition, a recent study showed that both amorphous and crystalline SiO_2_ particles had a similar effect on APP mRNA and protein in 1321N1 cells [42]. The physiological effect of the decrease in APP is currently unkown and actual formation and deposition of Amyloid β would require further in vivo studies.

An important role of connexins and maintenance of gap junctional intercellular communication (GJIC) between astrocytes was observed [19,22] and is involved in both cell invasion and resistance to oxidative stress [47,48]. Previous studies have shown that GJIC is relatively low in the 1321N1 cell line [42,49,50]. This is also indicated here by the small difference between CBX-treated and control cells. As the effect on proteins involved in apoptosis is small after 48 h, in addition to an increase in viability compared to the 24 h time point, it may be likely that the increase in GJIC is important for the exchange of survival signals. The duration of this would, however, be uncertain, as viability of the cells reduces after 72 h. The uptake of Lucifer Yellow was specific after scratching of the cell monolayer as cells further away from the scratches did not take up the dye. Thus, with the doses used here, disruption of the cell membrane as a result of SiMn exposure seems unlikely. An increase in GJIC in 1321N1 cells was also observed for amorphous and crystalline silica particles [42]. On the contrary, for example carbon nanotubes had a significant negative effect on GJIC and cell viability in a mouse fibroblastoma cell line showing that both the cell type and type of exposure is of great importance for GJIC [51].

In conclusion, the laboratory-generated dust that was collected on a filter after active oxidation of an industrial standard grade SiMn metal, contained 58% nano-sized particles and consisted mainly of Si and Mn. Analysis showed dose- and time-dependent effects on toxicological endpoints in astrocytoma cells, where higher doses gave reduced viability, changes in expression of apoptotic proteins, reduced expression of APP, and increased intercellular communication. It should, however, be noted that this study does not consider the complexity of particle translocation from the alveolar region of the lung to the circulation (air-blood-barrier), distribution to secondary organs and especially translocation to the brain through crossing the blood brain barrier. Therefore, the doses used in this study cannot be directly compared with exposure scenarios in the industry, and relevant exposure levels and accumulation in the brain remain to be established by in vivo studies.

## 4. Materials and Methods

### 4.1. Generation of the SiMn Dust

The SiMn dust employed in the current study was produced through active oxidation of an industrial standard grade SiMn metal. The molten metal was kept at 1500 °C while being exposed to an impinging air jet. The air flow was 3 L/min and synthetic air was used to ensure that moisture did not interfere with the oxidation. The dust was collected in a filter fabric of industrial standard. The technical details of the laboratory set-up and the thermodynamics and kinetics which govern the dust generation process has been described in detail in previous publications [2,3].

### 4.2. Preparation of the Dust for Characterization and Cell Culture Experiments

For dispersion of the dust, the NANOGENOTOX protocol for dispersion of nanomaterials was used with small adjustments [52,53]. Briefly, the dust was weighed and a solution of sterile-filtered 0.05% Bovine Serum Albumin (BSA; *m*/*v* in H_2_O) was added to obtain a stock solution of 1 mg/mL. BSA was used to get a well-dispersed dust. The solution was then vortexed and sonicated at 10% amplitude for 15 min using a probe sonicator (Sonifier 450S, Branson Ultrasonics, Danbury, CT, USA). A stock that was freshly prepared was used for each experiment. The doses used for cell culture exposures were 0, 2 × 10^−6^, 2 × 10^−5^, 2 × 10^−4^, 2 × 10^−3^, 0.02, 0.2 and 1.0 µg per cm^2^ surface area of the cell culture dish. The reason for choosing these doses were to test doses that were significantly lower than the doses reported in in vitro studies in the literature.

### 4.3. SiMn Dust Characterization

#### 4.3.1. Dynamic Light Scattering

The ZetaSizer Nano ZS (Malvern Instruments Ltd., Malvern, UK) was used to gain knowledge of the hydrodynamic size distribution of the dust after dispersion. In this regard, 1 mL of sonicated dust dispersion was added to a cuvette, left for 5 min on the bench, and was then left in the ZetaSizer apparatus for an additional 5 min before measurement over 10 cycles. In addition, to obtain information on the size distribution of the particles when dispersed in cell culture media, 1 mL of cell culture media containing the highest concentration of particles used for experiments was measured using ZetaSizer Nano ZS. For analysis of the data, ZetaSizer software (Malvern Instruments Ltd., Malvern, UK) was used. All data come from three independent measurements.

#### 4.3.2. Scanning Electron Microscopy

Both dry and dispersed dust was analyzed. Dry dust was prepared as follows: A Copper (Cu) TEM grid with holey carbon film (Holey Carbon film on Copper H7, Agar Scientific, Stansted, UK) was fixed onto a 25 mm PC-filter with a pore size of 1 µm. The particles were spread on an aluminum plate, and collected on the Cu TEM grid using open-face graphite-filled 25 mm filter holders with 50 mm extension tube (Gelman Air Monitoring Cassette, Gelman Sciences, Ann Arbor, MI, USA) through a 2 L/min nozzle. The particles on the Cu TEM grid were investigated without any further preparation. For characterization of dispersed dust, a volume corresponding to 100 µg was taken from a 1 mg/mL stock dispersed in 0.05% BSA which was sonicated as described above followed by filtering on a 47 mm Whatman Nuclepore polycarbonate filter with 15 nm pore size. A sputter coater (Cressington 208HR sputter coater, Watford, UK) was used to add a thin layer of platinum film on the filter. Thereafter, pieces of 10 mm × 10 mm were cut from the filter and gently fixed on aluminum specimen stubs with double-sided carbon adhesive discs. A Hitachi SU 6600 (Hitachi Industry and Control Solutions, Ltd., Ibaraki-ken, Japan) field emission scanning electron microscope (FE-SEM) equipped with a Bruker energy-dispersive X-ray (EDX) detector was used for SEM analysis. Accelerating voltage 15 keV and working distance 10 mm were used during EDX elemental analysis. Images of the particles were obtained by acquiring at slow scanning speed. Moreover, the specimens’ morphology and size were examined in the SEM.

### 4.4. Cells and Cell Culture

The human astrocytoma 1321N1 cell line was used for all cell culture experiments (Sigma-Aldrich, St. Louis, Missouri, MI, USA, catalogue no. 86030402). These are glial cells derived from a human brain astrocytoma that was initially isolated in 1972 as a sub clone of the cell line 1181N1 [54]. The passage number of the cells was kept below 30 and as a routine the cells were kept in a humidified 5% CO_2_ and 95% air incubator at 37 °C in Dulbecco’s Modified Eagle’s Medium (DMEM, Fisher Scientific, Hampton, New Hampshire, NH, USA) added 10% fetal bovine serum (FBS, Biochrom, Cambridge, UK), 50 U/mL penicillin and 50 μg/mL streptomycin (Thermo Scientific, Waltham, MA, USA).

### 4.5. Cytotoxicity Assay

For investigation of cell viability, 5000 cells were plated per well in triplicate in black 96-well plates with a transparent bottom (Nunclon, Thermo Scientific, Waltham, Massachusetts, MA, USA). After 24 h incubation, dispersed dust was added at the indicated doses. Then the medium was removed, and PBS was used to wash the cells for removal of excess particles. For the measurement of cytotoxicity levels, the Cell Counting Kit-8 (CCK-8) assay (Sigma-Aldrich, St. Louis, Missouri, MI, USA) was used according to the manufacturer’s instructions. After incubation at 37 °C for 1 h, absorbance as optical density (OD) was measured at 450 nm using a SpectraMax i3 (Molecular Devices, San Jose, California, CA, USA). OD was also measured at 750 nm as a reference wavelength for background detection. A standard curve with a known number of cells was established to calculate the number of cells in each well.

### 4.6. Quantitative PCR (qPCR) for Measurement of APP Gene Expression

The mRNA level of the neurodegenerative marker *Amyloid precursor protein* (*APP*) was measured by qPCR. Briefly, total RNA was extracted using RNA-Solv reagent (OMEGA bio-tek, Norcross, Georgia, GA, USA). cDNA from one µg of RNA was prepared using qScript cDNA synthesis kit (Quanta Biosciences, Beverly, Massachusetts, MA, USA) according to the manufacturers’ recommendations. qPCR was performed on a StepOne Real-Time PCR system (Applied Biosystems, Foster By, California, CA, USA) with Perfecta SYBR Green FastMix, ROX (Quanta BioSciences, Beverly, Massachusetts, MA, USA). For all the tested genes, pre-designed primers were purchased from Sigma-Aldrich (St. Louis, Missouri, MA, USA). A serial diluted internal standard served as a control for the qPCR reaction. Relative gene expression levels were calculated and normalized to the average expression levels of *β-actin*, *GAPDH* and *TBP*.

### 4.7. Detection of APP by Western Blot Analysis

Western blot analysis was as described previously [55]. Briefly, protein concentrations were measured using NanoDrop-8000 (Thermo Scientific, Waltham, Massachusetts, MA, USA). A total of 25 µg of each protein sample was resolved on AnykD Mini Protean TGX stain free gels (Bio-Rad Laboratories, Hercules, California, CA, USA) and transferred to a PVDF membrane (Bio-Rad Laboratories, Hercules, California, CA, USA). The Trans-Blot Turbo blotting system (Bio-Rad Laboratories, Hercules, California, CA, USA) was used for transfer. Antibodies used were as follows: APP (Cell Signaling Technology, Danvers, Massachusetts, MA, USA) and GAPDH (Santa Cruz Biotechnology, Dallas, Texas, TX, USA). Horseradish peroxidase conjugated antibodies (Cell Signaling Technology) were used prior to chemiluminescent detection (GE Healthcare, Chicago, Illinois, IL, USA). Results were quantified using Image J.

### 4.8. Apoptosis Protein Array

The expression levels of 35 proteins related to or involved in apoptosis were analyzed using the Proteome Profiler™ Human Apoptosis Array Kit (R&D Systems, Minneapolis, Minnesota, MI, USA). Following the manufacturer’s instructions cell lysis and protein detection were performed. 400 µg of cell lysate was applied to the protein array membranes. The protein arrays were developed on an AI600RGB imaging system (GE Healthcare, Chicago, Illinois, IL, USA) and the detected signal intensities for each protein were quantified with ImageQuantTL software (GE Healthcare, Chicago, Illinois, IL, USA).

### 4.9. Functional Assay of Gap Junctional Intercellular Communication (GJIC) by Scrape Loading

GJIC was determined by quantitative scrape loading [56]. 1321N1 cells were cultured on cover slips in 12-well plates (NUNC) and grown until 80–90% confluency. The cells were then exposed to the indicated doses of the dust for 24 and 48 h. Before scrape loading the confluent cell layer was washed twice with PBS. Then 1 ml of 0.05% Lucifer Yellow (Sigma-Aldrich, St. Louis, Missouri, MI, USA) dissolved in PBS *w*/*o* Ca^2+^ and Mg^2+^ was added to each well and the cell monolayer was cut with a surgical scalpel four times. Cells were incubated with Lucifer Yellow for 4 min and then washed with PBS four times before fixation in 3.7% formalin *o*/*n*. The following day the wells were washed with PBS two times before mounting with Mowiol. During the whole experiment, cells from one well were exposed to the gap junctional inhibitor carbenoxolone (CBX) (100 µM, Alfa Aesar, Haverhill, Massachusetts, MA, USA) as a control for dye uptake solely by cutting the cell layer. Fluorescence was observed using a laser scanning microscope (LSM 710, Zeiss, Oberkochen, Germany) with a magnification of 20x and photographs were taken with an AxioCam camera (Zeiss, Oberkochen, Germany). Ten images were taken for each exposure. The public domain NIH Image program was used for analysis applying the same settings for each measurement. The distance of diffusion of the dye away from the scalpel cut was used to determine the degree of GJIC.

### 4.10. Statistics

The data were analyzed in Sigma Plot 12.0 using T-test and non-parametric Mann-Whitney test as appropriate, and *p* < 0.05 was considered statistically significant.

## Supplementary Materials

Supplementary materials can be found at http://www.mdpi.com/1422-0067/20/3/740/s1.
