# Supplementary material for: Mechanisms of Toxicity of Industrially Relevant Silicomanganese Dust on Human 1321N1 Astrocytoma Cells: An In Vitro Study
Source: Int J Mol Sci. 2019 Feb 10;20(3):740. doi: 10.3390/ijms20030740 (PMC6386893; doi:10.3390/ijms20030740)
Supplement: Supplementary file 1 [file ijms-20-00740-s001.pdf]

	24 h			48 h		
	2E-5	2E-4	2E-3	2E-5	2E-4	2E-3
**Bad**	-1,06	-1,02	-1,06	1,04	1,07	1,04
**Bax**	1,31	-1,23	-1,05	**2,39***	-1,02	-1,05
**Bcl-2**	-1,03	1,09	-1,04	1,12	**1,21***	1,13
**Bcl-x**	**-1,50***	-1,16	**-1,72***	1,07	1,04	-1,08
**Pro-Caspase-3**	-1,16	1,10	1,00	1,12	1,56	1,72
**Cleaved Caspase-3**	-1,15	1,12	-1,05	**1,31***	**1,38***	1,04
**Catalase**	**2,07***	**2,93***	**2,70***	1,14	1,20	1,16
**cIAP-1**	-1,18	1,07	-1,06	1,02	**1,17***	1,07
**cIAP-2**	-1,12	1,14	1,08	1,03	1,07	1,01
**Claspin**	-1,22	-1,04	-1,09	-1,02	1,10	-1,01
**Clusterin**	-1,18	1,03	1,03	-1,01	1,17	1,03
**Cytochrome c**	-1,27	1,04	-1,08	-1,02	1,26	1,08
**TRAIL R1/DR4**	1,29	1,15	-1,03	1,23	**1,09***	1,11
**TRAIL R2/DR5**	1,05	1,03	1,03	1,13	**1,19***	**1,16***
**FADD**	-1,23	-1,16	-1,23	1,07	1,23	1,13
**Fas/TNFRSF6/CD95**	-1,19	1,00	-1,18	1,11	**1,32***	**1,31***
**HIF-1α**	-1,14	-1,10	-1,22	1,03	1,13	1,13
**HO-1/HMOX1/HSP32**	-1,15	-1,03	-1,14	1,07	1,17	1,03
**HO-2/HMOX2**	-1,32	-1,11	-1,20	-1,35	1,16	1,04
**HSP27**	-1,01	1,13	1,08	-1,02	1,10	1,05
**HSP60**	-1,11	1,02	-1,12	1,02	1,07	**1,11***
**HSP70**	-1,11	1,04	-1,16	1,06	1,09	**-1,10***
**HTRA2/Omi**	-1,25	1,00	-1,09	1,09	**1,22***	1,09
**Livin**	-1,08	1,02	-1,03	**1,11***	**1,17***	1,03
**PON2**	1,06	1,03	1,13	1,07	1,08	-1,02
**p21/CIP1/CDKN1A**	1,10	**1,17***	**1,25***	1,01	**1,41***	**1,13***
**p27/Kip1**	1,16	1,02	1,06	1,12	**1,13***	1,03
**Phospho-p53 (S15)**	**-1,39***	-1,14	-1,18	-1,05	1,08	1,03
**Phospho-p53 (S46)**	-1,18	-1,05	-1,15	1,08	**1,10***	1,08
**Phospho-p53 (S392)**	-1,23	-1,05	-1,19	-1,01	1,16	1,07
**Phospho-Rad17 (S635)**	-1,19	-1,06	-1,08	**-1,45***	1,15	1,01
**SMAC/Diablo**	-1,35	-1,16	-1,18	-1,15	1,09	-1,03
**Survivin**	-1,27	-1,19	**-1,25***	1,10	**1,23***	1,11
**TNF RI/TNFRSF1A**	**-1,43***	-1,25	**-1,33***	1,12	**1,15***	-1,03
**XIAP**	-1,09	-1,02	1,02	1,15	**1,32***	1,14

Supplementary Table S1. Expression levels of 35 proteins related to or involved in apoptosis were analyzed using the Proteome Profiler™ Human Apoptosis Array Kit. Numbers in bold with asterix are significant from the control. Results represent average quantification related to controls, *n*=3. * $P \leq 0.05$ indicates significant difference from control-treated cells obtained with nonparametric Mann-Whitney test.

Fold change > 1.5
Fold change 1.3 to 1.5
Fold change -1.3 to -1.5
Fold change > -1.5
